# Transient chromatin decompaction at the start of *D. melanogaster* male embryonic germline development

**DOI:** 10.26508/lsa.202302401

**Published:** 2024-07-11

**Authors:** Yi-Ru Li, Li Bin Ling, Angel Chao, Sebastian D Fugmann, Shu Yuan Yang

**Affiliations:** 1 Department of Biomedical Sciences, College of Medicine, Chang Gung University , Taoyuan, Taiwan; 2 Institute of Biomedical Sciences, College of Medicine, Chang Gung University , Taoyuan, Taiwan; 3 Department of Obstetrics and Gynecology, Linkou Chang Gung Memorial Hospital , Taoyuan, Taiwan; 4 Department of Nephrology, Linkou Chang Gung Memorial Hospital , Taoyuan, Taiwan

## Abstract

Embryonic germ cells develop rapidly to establish the foundation for future developmental trajectories, and in this process, they make critical lineage choices including the configuration of their unique identity and a decision on sex. Here, we use single-cell genomics patterns for the entire embryonic germline in *Drosophila melanogaster* along with the somatic gonadal precursors after embryonic gonad coalescence to investigate molecular mechanisms involved in the setting up and regulation of the germline program. Profiling of the early germline chromatin landscape revealed sex- and stage-specific features. In the male germline immediately after zygotic activation, the chromatin structure underwent a brief remodeling phase during which nucleosome density was lower and deconcentrated from promoter regions. These findings echoed enrichment analysis results of our genomics data in which top candidates were factors with the ability to mediate large-scale chromatin reorganization. Together, they point to the importance of chromatin regulation in the early germline and raise the possibility of a conserved epigenetic reprogramming-like process required for proper initiation of germline development.

## Abstract (precis)

Our genomics profiling of the earliest fruit fly germline revealed a brief window of major chromatin remodeling in the male germline right after zygotic activation and suggests reprogramming in the early stages of germline to be an evolutionarily conserved phenomenon.

## Introduction

Germ cells are specified during early embryogenesis in *Drosophila melanogaster* and within hours undergo several important developmental transitions that define much of its identity. Formation of germ cells relies on the maternal deposition of germplasm at the embryonic posterior, and multiple components of the germplasm are known to be critical, including Nanos (Nos) and Vasa (Vas), which are conserved as germline-specific factors across a wide range of species (Santos-Rosa et al, 2002). These components are needed to initiate the germline program, in part by delaying transcriptional activation with Polar granule component (Pgc) suppressing RNA polymerase II activity (Hanyu-Nakamura et al, 2008). Germ cells enter a new phase when transcription of the germline genome is activated (Van Doren et al, 1998), but the underlying mechanisms of this transition, critical to the sustainment of the germline program, are largely unknown. Of the major mechanisms that regulate gene expression and cellular differentiation, RNA-related processes are well represented in the germline of many species including fruit flies (Voronina et al, 2011). Transcriptional activation mediated by transcription factors (TFs) has been shown to be involved in the specification of mammalian germline (Irie et al, 2015), and though TFs acting analogously have not been found in *Drosophila*, our previous work has indicated transcriptional regulation to be an essential component to germline-biased gene expression (Li et al, 2021). Chromatin status could be part of such control, and in mammalian primordial germ cells, there are global DNA demethylation, reorganization of histone modifications, and other chromatin remodeling events that erase parental and somatic epigenetic marks to enable germline development (Ramakrishna et al, 2021). There have been no reports of similar events during embryonic germline development in the fruit flies.

Another important developmental milestone germ cells achieve during early development is to choose between the male and female fates, a decision that will result in two separate developmental paths that differ in gene expression and developmental timing among others. In several species including fruit flies, successful germline sex determination involves an external signal in addition to a germline-intrinsic component (Murray et al, 2010). In flies, the male somatic signal is supplied through the JAK/STAT pathway from the soma of the embryonic gonad (Wawersik et al, 2005), but the identity of the female signal is not known. Several factors have been shown to act within the germline to regulate its sex choice with the most notable examples being Sex lethal (Sxl) and PHD finger protein 7 (Phf7), which are able to induce male-to-female and female-to-male germline sex reversal, respectively (Hashiyama et al, 2011; Yang et al, 2012). However, these two factors are likely acting downstream of a yet unidentified sex switch responsible for reading the sex chromosome content in the germline.

In this study, we used single-cell ATAC sequencing (scATAC-seq) to survey chromatin accessibility of the embryonic fruit fly germline. Aided by two additional transcriptome datasets of single-cell RNA sequencing (scRNA-seq) and single-nucleus RNA sequencing (snRNA-seq), our analysis revealed that immediately after zygotic activation, the male germline, which initiates development, undergoes a phase of genome-wide chromatin decondensation that is repackaged shortly thereafter. Interestingly, several factors with the ability to alter chromatin structure and domains, such as the boundary element binding proteins Motif 1 Binding Protein (M1BP), Boundary element-associated factor of 32kD (BEAF-32), and the pioneer factor Zelda (Zld) (Liang et al, 2008), were found to exhibit germline enrichment in our datasets. These bring forth the idea that chromatin remodeling in the earliest germline could be an evolutionarily conserved process for resetting the germline epigenome required for their unique development.

## Results

### Epigenome and transcriptome profiling in the embryonic germline

**Figure 1.**
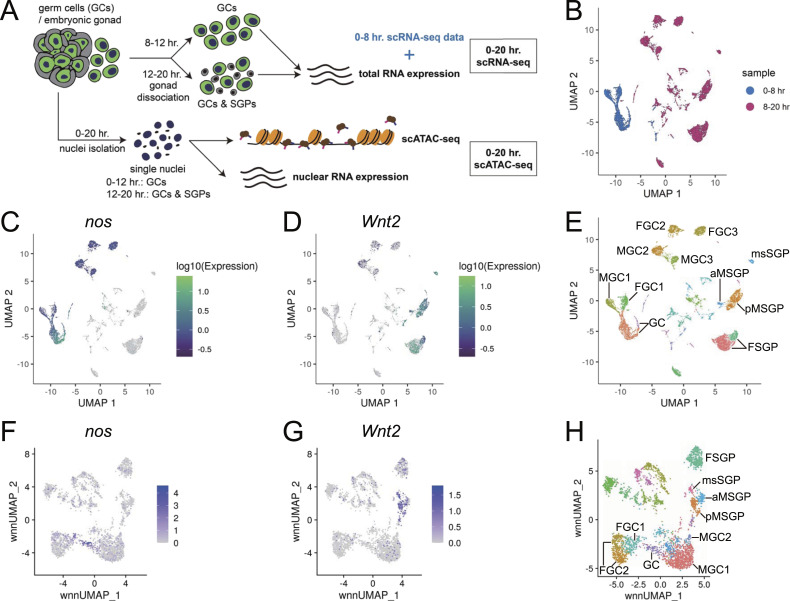
Identification of cell types in our scRNA-seq and scATAC-seq datasets. **(A)** Diagram depicting our experimental design. **(B)** Distribution of cell clusters after combining the 8- to 20-h scRNA-seq data (magenta) with the 0- to 8-h data we previously published (blue). **(C, D)** Expression patterns in the scRNA-seq dataset of *nos*, a germline-specific gene, and *Wnt2*, which is enriched in the embryonic somatic gonad. Color codes for expression levels are on the right of the plots. **(E)** Cell-type designations in the 0- to 20-h combined scRNA-seq dataset. GC, germ cells before zygotic activation; F/MGC1, F/MGC2, and F/MGC3, female/male germ cells at first, second, and third stages post-zygotic activation; F/aM/pM/msSGP, female/anterior male/posterior male/male-specific somatic gonadal precursors. **(F, G)** Nuclear expression patterns of the germline marker *nos* and somatic gonadal marker *Wnt2* in the multiome dataset. Color codes for expression levels are on the right of the plots. **(H)** Designation of cell types in the multiome dataset; abbreviation same as in (E) except that only two stages each for the female and male germ cells after zygotic activation were called (F/MGC1, F/MGC2).

To generate comprehensive coverage of the key molecular characteristics setting the early foundation for germline development, we acquired several single-cell genomics datasets for the fruit fly embryonic germline (Fig 1A). As we have previously performed scRNA-seq on 0- to 8-h germline (Li et al, 2021), we complemented this dataset with an 8- to 20-h collection that would complete the timeline covering embryogenesis. Embryonic germ cells coalesce with somatic gonadal precursors (SGPs) to form embryonic gonads around 10 h; thus, we performed sample collections for 8- to 12-h and 12- to 20-h time points separately. The 8- to 12-h collection involved simple mechanical disruption of the embryos to obtain germ cells as they were only loosely attached to the embryo, and the dislodged germ cells were then purified by FACS via green fluorescence because of the presence of the germline-specific *vas-GFP* transgene. For 12- to 20-h samples, embryo homogenization resulted in entire embryonic gonads being released from the embryos. The gonads, containing both germ cells and SGPs, were also FACS-sorted based on germline GFP expression. After isolation of the gonads, they were enzymatically digested to separate the germ cells and SGPs, and both cell types were included in the single-cell genomics experiments.

**Figure S1.**
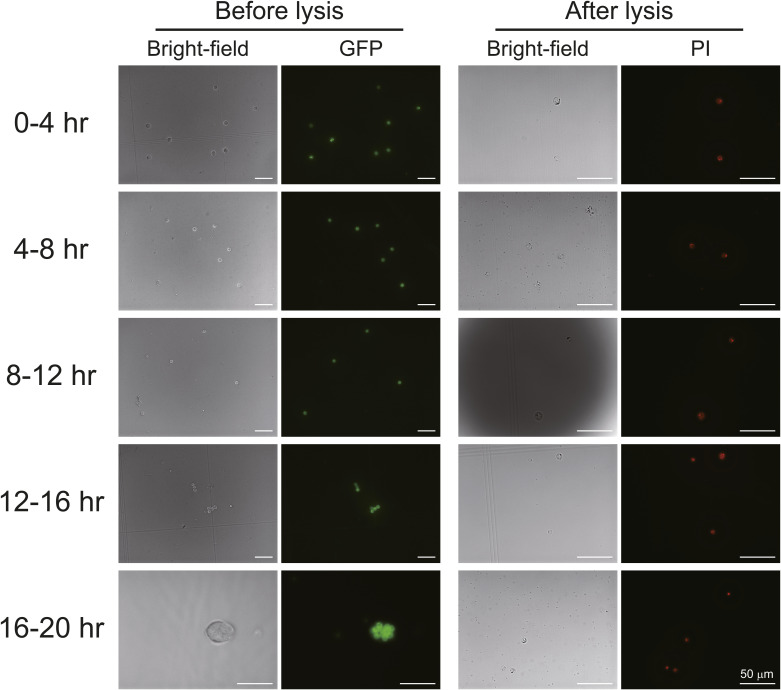
Quality control of nucleus preparations using embryos of different ages by microscopy. Germ cells (up until the 12-h time point) and gonads (12–20 h) before lysis exhibited GFP expression because of the *vas-GFP* transgene, and nuclei emitted red fluorescence after lysis because of propidium iodide (PI) staining. Scale bars are 50 μm.

In addition to scRNA-seq, we performed scATAC-seq on the embryonic germline to obtain chromatin accessibility profiles (Fig 1A). To obtain single nuclei for ATAC-seq, 0- to 12-h FACS-sorted single germ cells were lysed, whereas for 12- to 20-h samples, embryonic gonads were isolated by FACS and then lysed to obtain single nuclei of both germ cells and SGPs without having first dissociated the gonads into single cells (Fig S1). Besides scATAC-seq, RNA from these nuclei was also surveyed concurrently via the technique of snRNA-seq. The information from the RNA-seq of the single nuclei was essential for the determination of cell types in the multiome dataset composed of the scATAC-seq and snRNA-seq results.

**Figure S2.**
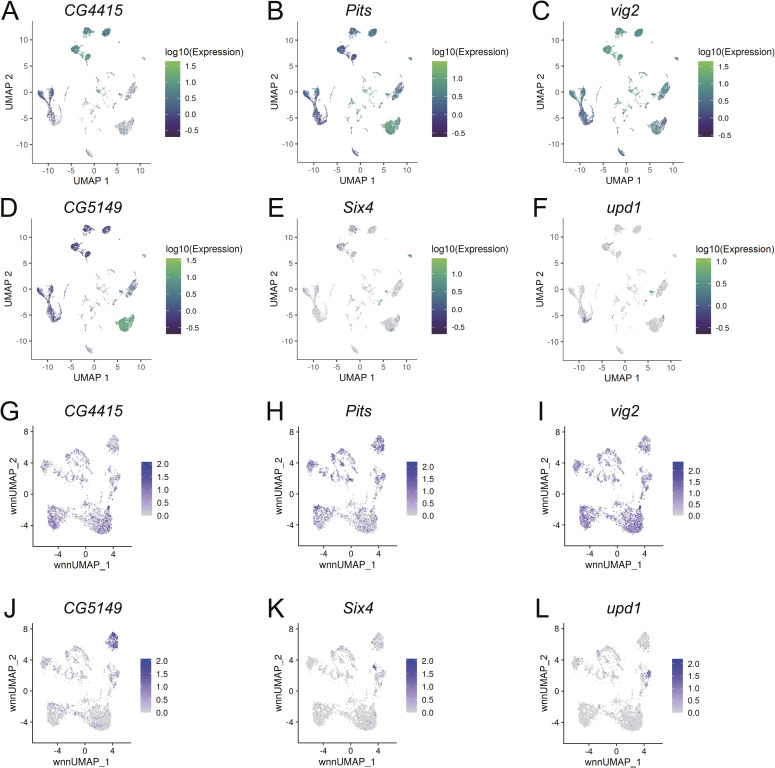
Expression profiles of key marker genes for delineating cell clusters in the genomics datasets. **(A, B, C, D, E, F, G, H, I, J, K, L)** The same marker genes were used for the scRNA (A, B, C, D, E, F) and multiome (G, H, I, J, K, L) datasets.** (A, G)** *CG4415* is a germline gene activated at zygotic activation. **(B, H)** *Pits* is a gene that is expressed more highly in the female germline than males after zygotic activation. **(C, I)** *vig2* is a gene that exhibits biased expression in the male germline compared with females after zygotic activation. **(D, J)** *CG5149* is a marker for FSGP. **(E, K)** *Six4* expression marks the msSGP population. **(F, L)** *upd1* expression is known to be enriched in male SGPs. Color codes for expression levels are on the right of plots.

To identify cell types and stages represented in the scRNA-seq data, we combined the results from our previous 0- to 8-h experiment with the new 8- to 20-h dataset (Fig 1B) (Li et al, 2021). This enabled us to use expression patterns of genes known to exhibit cell type and stage biases as identifiers for clusters (Figs 1C and D and S2A–F). For scRNA-seq, the merged 0- to 8-h and 8- to 20-h datasets gave rise to seven germline clusters and four SGP clusters (Fig 1E). Of the germline clusters, the one designated as “GC” represented cells before zygotic activation and sex differentiation, two key events that define the branch point of the large Y-shaped germline cluster, which was determined by our previous analysis for the 0- to 8-h dataset (blue cluster, Fig 1B) (Li et al, 2021). The remaining six germline clusters, all post-zygotic activation, consisted of three successive stages each for the male and female germline (designated as F/MGC1, F/MGC2, and F/MGC3, Fig 1E). For SGPs, one and three clusters respectively were found for females (FSGP) and males, and the latter included an anterior (aMSGP), a posterior (pMSGP), and a small cluster representing the male-specific SGPs (msSGP, Fig 1E) (DeFalco et al, 2003). For the multiome dataset, because it included snRNA-seq data, we were also able to use expression patterns of marker genes to identify all expected cell types (Figs 1F and G and S2G–L). A total of five germline clusters were called, one representing early, unsexed germ cells (GC) in addition to four sexed germline clusters, two for males and two for females (MGC1, MGC2, FGC1, and FGC2, Fig 1H). For SGPs, we identified one female cluster (FSGP) and three male ones, anterior, posterior, and male-specific (aMSGP, pMSGP, and msSGP, Fig 1H).

Several of the marker genes we used here showed expression biases rather than specificities, and some of the biases were modest, for instance, in *Protein interacting with Ttk69 and Sin3A* (*Pits*) and *vig2*, which were used for differentiating between female and male germline clusters after zygotic activation (Fig S2B, C, H, and I). However, their enrichment patterns in one cell type over another were reproducible as demonstrated previously in the 0- to 8-h dataset and reinforced in the 8- to 20-h dataset newly reported here (Li et al, 2021).

### Window of chromatin decondensation in the early male germline

**Figure 2.**
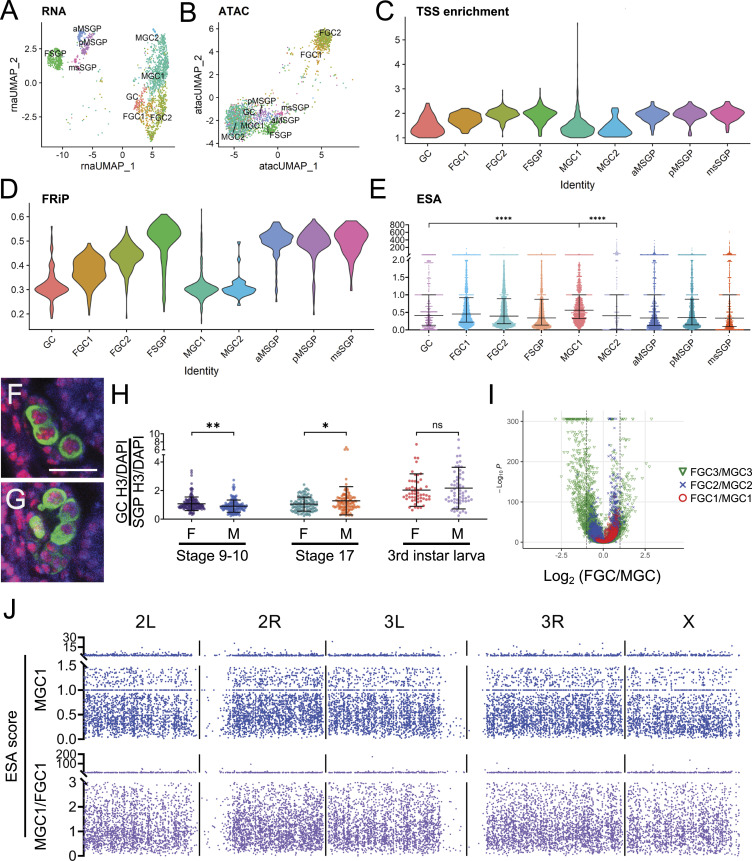
Chromatin structure of the embryonic germline. **(A)** Clustering pattern using the snRNA-seq data alone. **(B)** Clustering result with the scATAC-seq data alone. For (A, B), cell-type designations are the same as for the combined multiome dataset. **(C)** Violin plots of transcription start site enrichment values for all genes in individual clusters. **(D)** Violin plots of FRiP values for all genes within each cluster. **(E)** Box-and-whisker plots displaying end-to-start accessibility values for all genes from individual clusters. The three horizontal bars for each cluster from top to bottom mark the 75%, 50%, and 25% values. **** indicates *P* < 10^−5^. **(F, G)** Representative images of histone H3 staining (red) in the embryonic testis (F) and ovary (G). Samples were also stained with α-Vas (green) and DAPI (blue), and the two images are of the same magnification with the scale bar in (F) indicating 20 μm. **(H)** Quantitation of H3 signals in the female and male germline at three developmental stages, stage 9–10, stage 17, and third-instar larvae, for which only germline stem cells were scored. The three horizontal bars for each cluster mark from top to bottom the 75%, 50%, and 25% values. * and ** indicate *P* < 0.01 and 0.001, respectively; ns, not statistically significant. **(I)** Overlaying volcano plots depicting the female-to-male expression ratios on the x-axis for the three germline stages past zygotic activation with *P*-values plotted on the y-axis. Red, FGC1/MGC1; blue, FGC2/MGC2; green, FGC3/MGC3. **(J)** End-to-start accessibility values for individual genes plotted across all chromosomes, either for the MGC1 cluster (blue, top) or as relative values between MGC1 and FGC1 (purple, bottom).

The clustering pattern of the combined multiome dataset was quite similar to that of the scRNA-seq data in regard to the relative positions of clusters (Fig 1E and H), and this was also the case when only the snRNA-seq data within the multiome dataset were used to perform clustering (Fig 2A). In comparison, clustering with the scATAC-seq data alone led to a major separation of the female germline clusters (FGC1 and FGC2) from the other three germline clusters (GC, MGC1, and MGC2, Fig 2B). This suggested that although gene expression patterns among all germline clusters shared many similarities, there were likely substantial differences in chromatin accessibility between germline clusters, and indeed, such differences were evident based on several common parameters for assessing ATAC-seq results. Typically, ATAC-seq reads are concentrated around transcription start sites (TSSs) as these areas are less occupied by nucleosomes to allow transcriptional activation, and TSS enrichment values of most cell types are greater than 1 (Yan et al, 2020). In our scATAC-seq dataset, the SGP clusters and FGC clusters exhibited the expected TSS enrichment values, whereas the values for GC, MGC1, and MGC2 were lower (Fig 2C). Another parameter, fraction of reads in peaks (FRiP), also reports the pattern of ATAC-seq reads in a cluster, and they are commonly greater than 0.3 (ENCODE Standards). FRiP values for SGP and FGC clusters matched expectations, whereas those for GC, MGC1, and MGC2 were again lower (Fig 2D).

**Figure S3.**
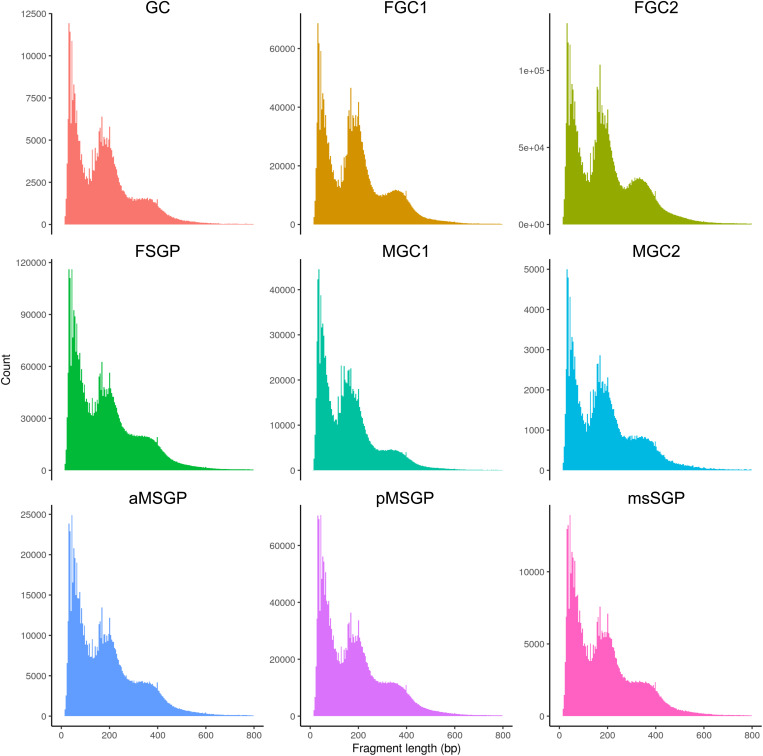
Distribution of scATAC-seq fragment lengths of various clusters by plotting the number of reads (y-axis) of a given size (x-axis).

These unexpected patterns suggested that the reads in GC, MGC1, and MGC2 were not be as focused as in other clusters, but it is important to rule out technical issues being the underlying cause of these results. The nuclei of the MGC1 and MGC2 clusters were collected and prepared together with those of the female GC clusters and all SGP ones, and the TSS enrichment and FRiP values outside of MGC1 and MGC2 were fully in line with standards (Fig 2C and D); if there were technical problems, nuclei for different clusters should all be affected. In addition, the scATAC-seq fragment sizes for GC, MGC1, and MGC2 were comparable to other clusters and consistent with expected patterns (Fig S3). Taken together, we considered the atypical values of TSS enrichment and FRiP in the MGC1 and MGC2 clusters unlikely to be artifacts but are reflecting the chromatin structure in these early male populations in being more open and different from other cell types and stages. This is a possibility that would not be unreasonable given that these early male germ cells were in a distinct phase: they were only a few hours post-specification and were undergoing major gene expression changes to initiate unique developmental processes.

**Figure S4.**
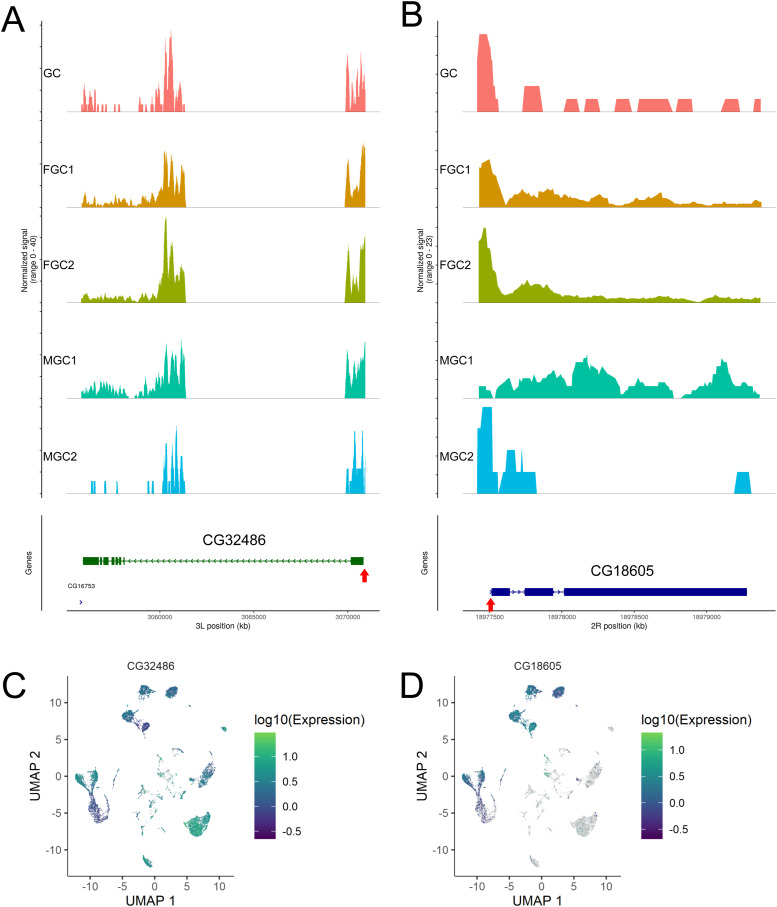
Profiles of two examples of zygotically activated germline genes, *CG32486* and *CG18605*. **(A, B, C, D)** Distributions of scATAC-seq reads in different clusters are as indicate (A, B) and gene expression in the scRNA-seq dataset are shown for comparisons (C, D). Red arrows in A and B indicate TSS positions.

These observations prompted us to examine the distribution of ATAC-seq reads on example genes to investigate the origin of differences in TSS enrichment and FRiP values for various clusters. For FGC1 and FGC2, most genes we examined had ATAC-seq read patterns concentrated around TSSs as in two example genes given here, *CG32486* and *CG18605* (Fig S4A and B). Both genes were up-regulated at zygotic activation with *CG18605* showing much greater germline specificity (Fig S4C and D). Interestingly, ATAC-seq reads in *CG32486* in the GC, MGC1, and MGC2 clusters exhibited strong TSS enrichment (Fig S4A), but for *CG18605*, the extents of read concentration around TSSs in GC and MGC2 clusters were less than those in FGC clusters, and in MGC1, the reads were widely spread across the entire gene body (Fig S4B). This pattern could be an outlier, or it can be reflective of the chromatin state of most loci for cells of the MGC1 cluster; thus, it was important to quantitatively examine the distribution of ATAC-seq reads for all genes. For this purpose, we introduced an “end-to-start accessibility” (ESA) score that can be calculated for each gene for all cells in a cluster. It is defined as the ratio of the number of reads within ±250 bp of the ends of transcripts to the number of reads within ±250 bp of TSSs (Fig 2E). Higher average ESA values for a cluster would indicate broader distribution of ATAC-seq reads, whereas lower scores would be expected for clusters whose reads are focused around the 5′ end of genes. Using this parameter, we indeed saw that MGC1 had significantly higher ESA scores compared with GC and MGC2 (Fig 2E), the two clusters of germline stages immediately abutting that of MGC1. This finding was consistent with the gene-by-gene patterns we observed for ATAC-seq and revealed that chromatin accessibility was particularly deconcentrated in MGC1, suggesting altered nucleosome density and patterning in this cell stage.

To corroborate the finding that chromatin structures in different germline clusters were distinct, we performed immunofluorescence staining for histone H3 in the developing germline as a proxy for nucleosome density (Fig 2F and G). We were able to observe dynamic sex and stage differences in histone H3 staining intensities of the developing germline (Fig 2H). In stage 9–10 germ cells, male H3 staining was lower in average intensity compared with females, but this trend is reversed in late-stage (stage 17) embryonic germline and in the third-instar larva, the stage at which female germline start to develop. These changes corresponded to differences in the timeline of male versus female germline development: the male germline initiates division and differentiation in late embryogenesis, whereas female ones do not start developing until the third-instar larval stage. This difference in developing timing was reflected in our scRNA-seq results: in the first stage post-zygotic activation, a handful of genes exhibited female-biased expression, but by the third stage, sex-biased germline genes were overwhelmingly male-biased (Fig 2I). The immunostaining results of histone H3 suggested that nucleosome density in the germline is developmentally regulated to support stage-specific and sex-specific gene expression regulation.

We further wondered whether the unique chromatin accessibility and nucleosome patterns in MGC1 are arranged in large domains. To address this point, we plotted ESA values of all genes along individual chromosomes for the MGC1 cluster; we also graphed the relative values of MGC1 ESA scores divided by those of FGC1 to eliminate non-specific locus variations. For both plots, the ranges of values appeared to be randomly distributed along all chromosomes without any stretches that were consistently higher or lower (Fig 2J). This indicated that the more evenly distributed chromatin accessibility in MGC1 is not a product of specific domains being regulated differently but rather a general feature of the entire genome. We will note that not all genes in cells of the MGC1 cluster exhibited the evenly spread-out ATAC-seq read pattern such as that observed in *CG18605* (Figs 2E and S4A and B). This could be a result of the epigenome reorganization process having occurred rapidly, and that of the chromatin structure at various loci not being fully synchronized. Our data here did not address the physiological significance of the unique and transitional nature of chromatin patterns in MGC1 nor the mechanisms underlying this phenomenon. However, these observations suggested that the early male germline undergoes a short period during which epigenome reorganization occurs; potential implications of this feature are presented in the Discussion section.

### Involvement of long-range chromatin regulators in the embryonic germline

The establishment and maintenance of the unique germline lineage after zygotic activation requires the expression of genes specific to the germ cells. The most common strategy for cell type–specific gene expression is through TFs regulating gene expression in a cell type–restricted manner. Post-transcriptional mechanisms can also alter the steady-state RNA levels of a gene. Our previous scRNA-seq analyses indeed indicated that mechanisms beyond regulating transcriptional activation such as promoter-proximal RNA polymerase II pausing and controlling RNA stability contribute to the establishment of a germline-biased gene expression program during zygotic activation (Li et al, 2021). Nonetheless, there is clearly a set of genes that are being transcriptionally activated in a germline-enriched manner.

To investigate how germline-biased transcriptional induction is achieved, we wanted to use our datasets to identify consensus DNA motifs enriched on genes that are selectively transcribed in the embryonic germline compared with soma, and such analysis would require data on transcriptional activities of genes in both the germline and soma. For the germline, we reasoned that our snRNA-seq dataset was a better resource than the scRNA-seq data as the latter measured steady-state mRNA levels, whereas profiles of nuclear RNA would more closely follow levels of ongoing transcription. For somatic transcriptional levels, we referenced a global run-on sequencing (GRO-seq) study that profiled nascent transcripts in 3- to 3.5-h-old embryos (Saunders et al, 2013). The ratios of snRNA-seq for various germline clusters from our dataset to GRO-seq for soma would approximate how much transcriptional activity of each gene is biased toward the embryonic germ cells. Though the age of embryos in the GRO-seq dataset is not identical to our germline data, it is a time point shortly after zygotic activation in the somatic embryo has occurred; thus, we consider it to be a reasonable comparison with the data we acquired for the embryonic germline right after its own zygotic activation.

**Figure 3.**
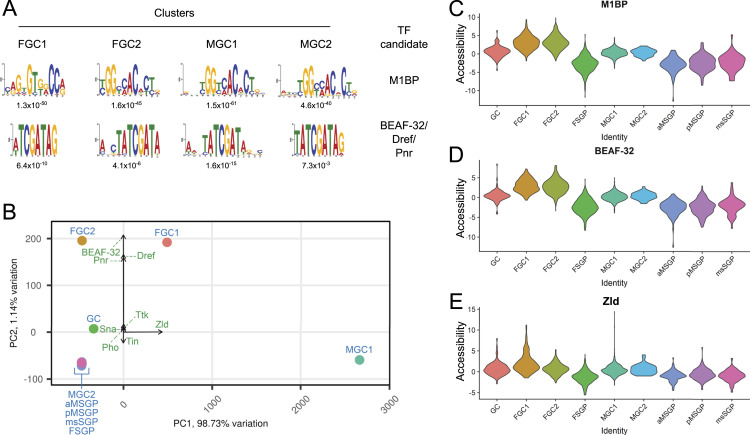
Profiles of top candidate factors with the ability to regulate gene expression. **(A)** Two most enriched DNA binding motifs in the promoter regions of the top 150 genes exhibiting strongest biases in gene expression for each of the indicated germline clusters compared with that of embryonic soma. The factor most likely associated with each sequence is annotated on the right, and the *E*-values for each motif are indicated below. **(B)** PCA plot demonstrating the relationships of overall TF binding site chromatin accessibility between clusters, which are designated with solid circles and labeled in blue. The lengths and directions of arrows represent the contribution levels and directions, respectively, of the most significant TFs, whose identities are labeled in green. **(C, D, E)** Violin plots displaying accessibility scores of the binding sites of three of the TFs that exhibit the strongest cluster-biased patterns. **(C)** M1BP, (D) BEAF-32, and (E) Zld.

With the above formula, we calculated the germline-to-soma transcription ratios of all genes using snRNA-seq expression values from the four germline clusters post-zygotic activation (MGC1, MGC2, FGC1, and FGC2, Table S1). The top 150 genes from each of the resulting lists were used in the searches for common DNA sequences by performing motif discovery analysis in the 500-bp regions upstream of the TSSs. Once identified, the motifs were then matched to known binding sites of TFs within the footprintDB database to reveal the most likely associated factor (Sebastian & Contreras-Moreira, 2014). The top two binding sites found for all four post-zygotic activation germline clusters were the same: those of M1BP and BEAF-32/Dref/Pnr (Fig 3A and Table S2). The consensus motifs of BEAF-32, DNA replication-related element factor (Dref), and Pannier (Pnr) are very similar; thus, they likely represented the same pair of the binding site and associated TF. M1BP has been reported to act as a TF and a boundary element binding protein (Bag et al, 2021). Interestingly, BEAF-32 is also known to be associated with boundary elements (Gerasimova & Corces, 1996). Boundary elements are able to regulate chromatin structure at a larger scale; thus, the potential involvement of M1BP and BEAF-32 in germline gene activation implied the presence of broad-scale chromatin organizational mechanisms in regulating germline gene expression. Aside from the binding sites of M1BP and BEAF-32, several other motifs were found to be over-represented in genes exhibiting the strongest germline expression biases (Table S2). Thus, germline-specific expression is most likely established via multiple different strategies, some potentially acting over longer distances whereas others functioning on a set of nearby loci.

Table S1. Comparisons of gene transcriptional activity using snRNA-seq expression values in the embryonic germline and GRO-seq gene body expression values in the embryonic soma.

Table S2. Common motifs discovered in genes enriched at the transcriptional level in the indicated germ cell populations post-zygotic activation.

Our data on chromatin accessibility provided an additional angle from which we can investigate the contributions of TFs and chromatin regulators. We determined accessibility for all sites of all TF binding motifs within individual clusters and performed PCA to determine overall similarities in TF accessibility between different clusters (Fig 3B and Table S3). The female germline clusters (FGC1 and FGC2) were located near each other but separated from other germline clusters. Intriguingly, the MGC1 cluster was positioned distinctly and far away from all others, whereas MGC2 was located very close to the SGP clusters (Fig 3B). The uniqueness of MGC1 TF accessibility compared with its subsequent developmental stage, MGC2, whose pattern more closely resembled those of somatic clusters, suggested that MGC1 was undergoing a transitional phase before its chromatin profile converged with a more typical state observed in the embryonic somatic gonad as manifested by MGC2. These implications were reminiscent of what ESA scores revealed regarding the chromatin status of the male germline clusters. Together, our analyses suggested that the initiation of the male germline program requires a broad genome-wide reorganization at the chromatin level.

Table S3. Accessibility indices of TFs in the footprintDB database in different clusters.

**Figure S5.**
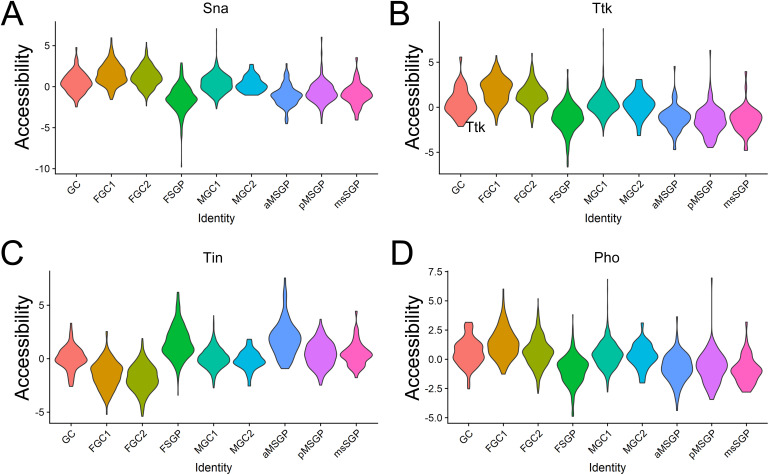
Graphs of accessibility indices by cluster for four TF binding sites that exhibited strong cluster-biased patterns and were found to be key differentiating factors between clusters based on overall TF accessibility (Fig 3B). **(A, B, C, D)** Snail (Sna), (B) Tramtrack (Ttk), (C) Tinman (Tin), and (D) Pleiohomeotic (Pho).

We further looked into accessibility indices of the individual TF binding site and were excited to find both M1BP and BEAF-32 as factors whose binding sites were among those exhibiting the greatest accessibility differences between clusters (Table S3). In fact, BEAF-32 appeared to be the biggest contributor to the separation of the female germline clusters from others (Fig 3B). Accessibility differences could also be visualized by graphing values of the individual TF for each cluster side by side (Figs 3C–E and S5A–D). For both M1BP and BEAF-32, accessibility indices of their binding sites were higher in all germline clusters compared with somatic ones, but there was another level of difference, and it was between the values for FGC clusters and other germline ones (Fig 3C and D). These suggested that M1BP and BEAF-32 are important for all germline clusters, but their roles and effects in various stages of germ cells are different.

Yet, another interesting binding site exhibiting accessibility bias was that of Zld (Fig 3E and Table S3), the pioneer factor critical for zygotic activation in the fruit fly embryonic soma (Liang et al, 2008). Pioneer factors are also known to direct reorganizations of chromatin domains; thus, Zld could be part of the mechanism alongside M1BP and BEAF-32 to direct chromatin structural changes that are necessary for the activation of germline gene expression.

### Partial dosage compensation of the germline X chromosome at the gene expression and chromatin level

Dosage compensation is a common process that cells between the two sexes employ to equalize the expression of genes residing on the sex chromosome as their copy numbers are different between cells of the two sexes. In the soma of fruit flies, the complex that mediates the up-regulation of the single male X chromosome so that X chromosome genes in male and female cells can be expressed at similarly optimal levels has been long known and is initiated by Sxl, which also controls somatic sex determination (Conrad & Akhtar, 2012). This complex does not operate in the germline, and the emerging picture is that dosage compensation of X chromosome genes in the germline is most likely dynamic and stage-dependent (Rastelli & Kuroda, 1998; Deng et al, 2011; Mikhaylova & Nurminsky, 2011; Meiklejohn & Presgraves, 2012; Vibranovski et al, 2012; Mahadevaraju et al, 2021).

Embryonic germline dosage compensation can be examined by calculating the relative gene dosage between male and female germline. If the expression level of a gene is the same between the male and female germline, the sex expression ratio would be 1, which is expected for autosomal genes. If there is complete dosage compensation in the germline, the same ratio would be expected for X chromosome genes. In contrast, the expected female-to-male expression ratio for X chromosomal genes would be 2 in the absence of dosage compensation as female cells carry two copies of the X chromosome as compared to just one in males. We had the data in the 0- to 8-h and 8- to 20-h scRNA-seq results to cover 12 h past the point of zygotic activation, the time during which dosage compensation, if it exists in the germline, should be enacted. For this analysis, we included the genes designated as being zygotically activated in the germline based on gene expression comparisons between the second germline stage post-zygotic activation (averages for FGC2 and MGC2) and the GC cluster (Table S4).

Table S4. List of zygotically activated genes in the germline determined by comparing the average expression of genes in the MGC2 and FGC2 clusters with that in the GC clusters.

**Figure 4.**
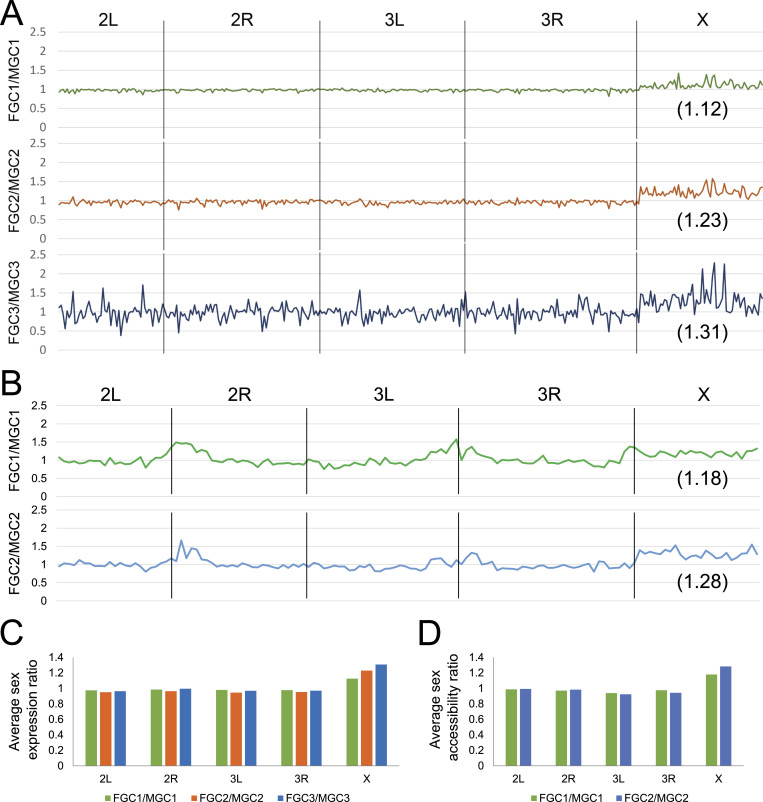
Genome-wide comparisons of female-to-male gene expression and chromatin accessibility for the determination of germline dosage compensation. **(A)** Female-to-male gene expression ratios across autosomes and the X chromosome for the three germline stages past zygotic activation from the scRNA-seq dataset. **(B)** Female-to-male accessibility ratios for autosomes and the X chromosome for the two germline stages post-zygotic activation from the scATAC-seq dataset. For both (A, B), the numbers in parentheses indicate the average of all ratios on the X in the respective germline stages. **(C)** Averages of female-to-male expression ratios for genes on individual chromosomes in the three germline stages based on the scRNA-seq dataset. **(D)** Averages of female-to-male accessibility indices separated by chromosomes in the two successive germline stages post-zygotic activation based on the scATAC-seq data.

After determining female-to-male gene expression ratios of the zygotically activated genes in the three successive germline stages post-zygotic activation, several trends were evident. First, sex expression ratios for autosomes were close to 1 right after zygotic activation, but they deviated more frequently and further from 1 as they progressed along developmental time (Fig 4A), indicating that expression differences between males and females widen as germ cells develop. However, the averages for autosomal genes remained very close to 1 (Fig 4C). For the X chromosome, average gene expression was higher in females than in males, but the female-to-male ratios were far less than 2 (1.12–1.31 for the three stages, Fig 4A and C). This moderate X chromosome–wide female-biased expression was observed immediately after zygotic activation in the germline, and it persisted throughout embryogenesis. These clearly suggested the presence of germline X chromosome gene dosage compensation, but the effectiveness of this mechanism is not 100%.

**Figure S6.**
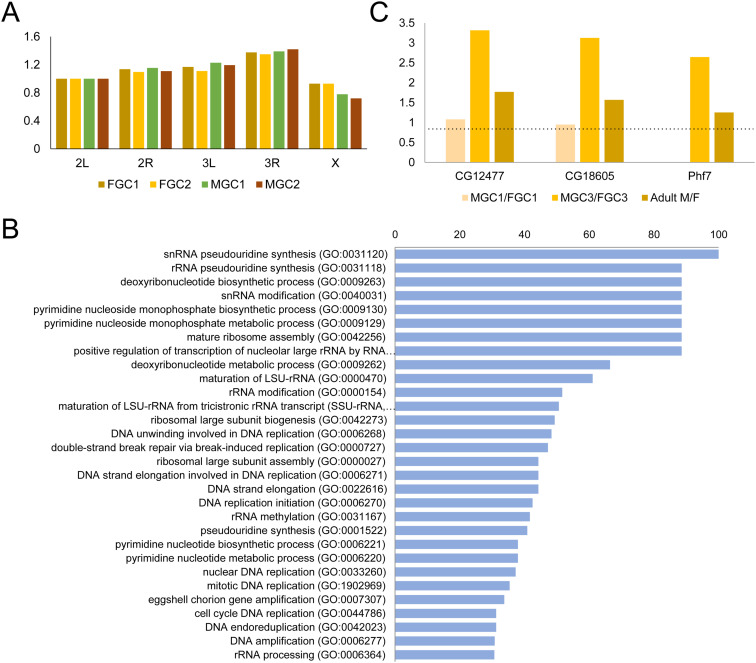
Characterization of sex-biased features in the embryonic germline. **(A)** Comparisons of total scATAC-seq read counts for each chromosome in the four germline clusters post-zygotic activation. The numbers are normalized to the 2L values. **(B)** GO-term analysis results of the 100 most male-biased genes in the third germline stage post-zygotic activation graphed with decreasing fold enrichment levels. **(C)** Male-to-female expression ratios of three male-biased TFs through germline development including the first and third germline stages post-zygotic activation in our scRNA-seq data and in adults. The dotted horizontal line marks the position of 1.

We further addressed germline dosage compensation at the level of chromatin accessibility as dosage compensation is commonly regulated in part by altering chromatin structure in a sex-specific manner, including in the fruit fly soma (Samata & Akhtar, 2018), and this is an aspect that has not been investigated in germline dosage compensation. Our scATAC-seq results were able to provide the answer to whether X chromosome accessibility reflects the presence of dosage compensation observed at the level of gene expression. We calculated “accessibility indices” for every 1-Mb region along all chromosomes, and these values would reflect how open genomics regions were based on the number of ATAC-seq reads mapping to each window. For autosomes, we found that the female-to-male ratio for accessibility indices of germline clusters hovered around 1 (Fig 4B and D), consistent with there being no sex biases in gene expression levels on autosomes. In comparison, the female-to-male ratios of accessibility indices for the X chromosome were higher than 1 but clearly less than 2 (Fig 4B and D), a finding comparable to what was found at the gene expression level. The compensatory effect of chromatin accessibility on X chromosomes between male and female germline could be a result of the female X chromosomes being less accessible or the male X becoming hyperaccessible. To address this point, we counted the total number of scATAC-seq reads mapping to the X chromosomes in both male and female germ cells. In female germ cells, the tallies, compared with those for autosomes, were proportional to their chromosomal lengths (Fig S6A), indicating that the female X chromosome has not become less accessible. For the male clusters, X chromosome read tallies were only slightly reduced compared with those for females even though male germ cells had just half the number of X chromosomes compared with females. This makes the number of reads per X chromosome in males much higher than that in females, indicating that the male X chromosomes do have increased accessibility. This characteristic likely enables higher X chromosome gene expression to help achieve dosage compensation.

Overall, our results suggested that there is partial germline dosage compensation occurring at both the gene expression and the chromatin structure level, and that the male X chromosome exhibits enhanced expression in part via increased accessibility to match the double dose of the gene expression of the two X chromosomes in the female germline.

### Sex-specific gene expression in the embryonic germline

The choice of proper sex is another critical developmental decision early germ cells must make, and sex-biased gene expression is most likely the first sign of sexual dimorphism as such differences should precede physiological differentiation. By examining sex-enriched gene expression through the three progressive stages of germline in our scRNA-seq dataset, we were able to uncover several waves of sex-biased gene expression patterns (Table S5). The earliest wave that coincides with zygotic activation in the germline was largely female-biased (Fig 2I) (Li et al, 2021). This initial over-representation of female germline–biased genes quickly gave way to the reverse trend a few hours later in which sex-biased genes became dominated by male ones (FGC3 versus MGC3, Fig 2I and Table S5). When we examined the types of genes that were male-biased during late embryogenesis, the majority were involved in housekeeping functions such as cell division and basic metabolism (Fig S6B). This is in line with the fact that male germ cells start to divide in stage 15, whereas female ones are quiescent. Nonetheless, there were a few other male germline–biased genes that stood out. One of them was *no child left behind* (*nclb*), a factor previously reported as male germline-biased during embryogenesis and that regulates germline stem cell maintenance (Casper et al, 2011). In addition, two genes that encode putative zinc finger family TFs, *CG18605* and *CG12477*, were also high on the male-enriched list. These two genes also exhibited male-biased expression in the adult germline (Fig S6C), suggesting male-specific germline roles for these two factors during development. As a point of comparison, we examined the best-known male germline factor, *Phf7*, and found it to also exhibit a male expression bias in the late germline stage of our data (Fig S6C). It was 2.64-fold more highly expressed in the male germline compared with females, placing it only as the 170th most male-biased gene at this stage (Table S5).

Table S5. Sex-enriched genes in the three germline stages past zygotic activation as calculated by DESeq2.

**Figure 5.**
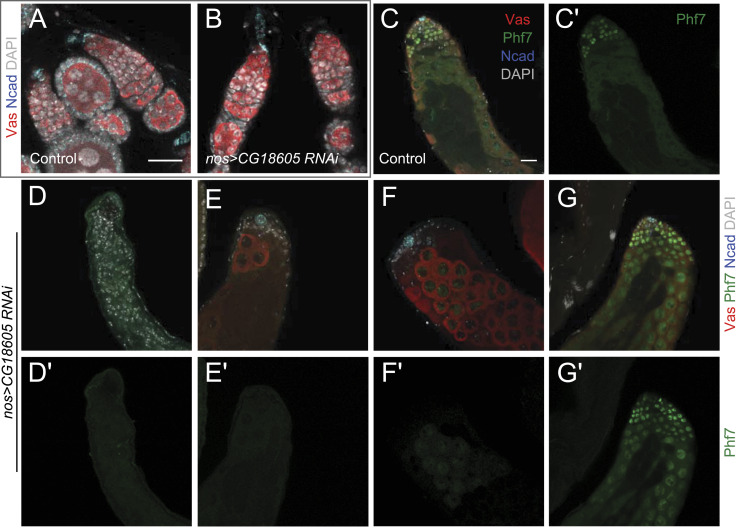
RNAi knockdown analysis of the male embryonic germline–biased gene CG18605 in the testis and ovary by immunofluorescence staining. **(A, B)** Control (*nos-Gal4/+*, (A)) and *CG18605* germline knockdown (*nos-Gal4/UAS-CG18605 RNAi*, (B)) ovaries stained with antibodies recognizing Vas (red) and N-cadherin (blue) in addition to DAPI (gray). **(C, D, E, F, G)** Images of control (*nos-Gal4/+*, (C)) and *CG18605* germline knockdown (*nos-Gal4/UAS-CG18605 RNAi*, (D, E, F, G)) testes stained with antibodies recognizing Vas (red), Phf7 (green), and N-cadherin (blue) in addition to DAPI (gray). **(C′, D′, E′, F′, G′)** Displays of the Phf7 channel alone from the same images of (C, D, E, F, G). **(D, E, F, G)** *CG18605* knockdown in the male germline resulted in a range of phenotypes including complete loss of germline (D), sparse spermatocyte (E), loss of spermatogonia (F), and abnormal Phf7 expression (G). All ovaries and all testes are of the same magnification with the scale bars in control images showing 20 μm.

To provide functional validation to our scRNA-seq results, we carried out germline-specific RNAi knockdown of *CG18605* to examine whether the male-enriched gene expression would correspond to male germline-specific physiological roles. In the female germline, suppressing *CG18605* expression led to minimal differences in the ovary compared with controls (Fig 5A and B). In contrast, male germline knockdown of *CG18605* resulted in strong germline phenotypes (Fig 5C–G). In nearly a quarter of such testes, there was a loss either of all spermatogonia (11% of all testes, Fig 5D) or of all germline (another 11%, Fig 5E and F). In the testes that appeared to have near-normal germline numbers and organization (78%), there was the ectopic expression of Phf7 in the nuclei of spermatocytes when normal Phf7 expression is abruptly down-regulated at the end of spermatogonia (Fig 5G compared with Fig 5C). In summary, disruption of *CG18605* expression caused male germline defects with near 100% penetrance, and though a variety of phenotypes were observed, it is possible that the origins of the defects were the same but manifested differently depending on the strength of *CG18605* knockdown in individual tissues. As *CG18605* is predicted to encode a TF, one possibility is that its down-regulation resulted in the misexpression of other male germline factors such as Phf7, thereby causing developmental defects in the male germline, in particular in the undifferentiated stages. These results indicated that *CG18605*, being a gene exhibiting male germline–biased expression, does function in a male germline–specific manner, and this validated our single-cell genomics profiling in being able to uncover important biological effectors for germline sex development.

### Comparisons between male and female SGPs

**Figure S7.**
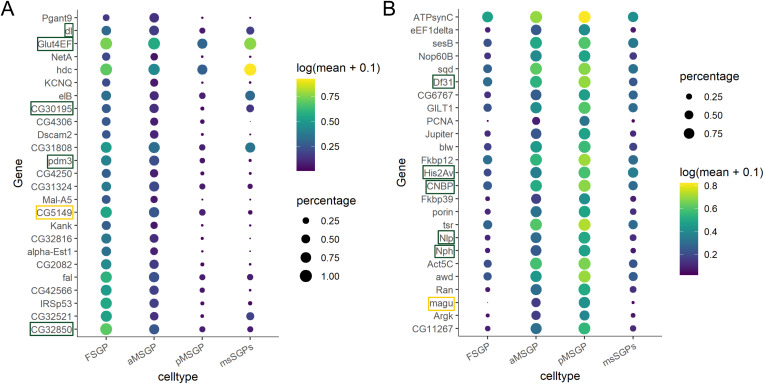
Sex-biased gene expression in the embryonic somatic gonadal precursors (SGPs) determined by comparing FSGP1 with aMSGP using DESeq2. **(A, B)** Top 25 genes exhibiting highest female-to-male (A) and male-to-female (B) gene expression biases in SGPs. Those that encode TFs are highlighted by green boxes. Two genes, *CG5149* and *magu*, have previously been published as being sex-biased in SGPs and are highlighted in yellow. Codes for expression levels are on the right.

The samples we collected for our single-cell experiments included embryonic SGPs, cells whose primary role is to nurture germline development. To expand on our knowledge of what distinguishes the male and female SGPs, we investigated the genes that showed the greatest sex biases in the early SGP populations (FSGP versus the average for all MSGP clusters) (Fig S7A and B). Of the top 25 candidates for SGPs of either sex, two genes known to be male- and female-biased, respectively, *magu* and *CG5149* (Casper & Van Doren, 2009; Zheng et al, 2011), were found, confirming the robustness of our data. Several TFs were also on the list and would be candidates for factors that regulate sex-specific SGP development.

**Figure 6.**
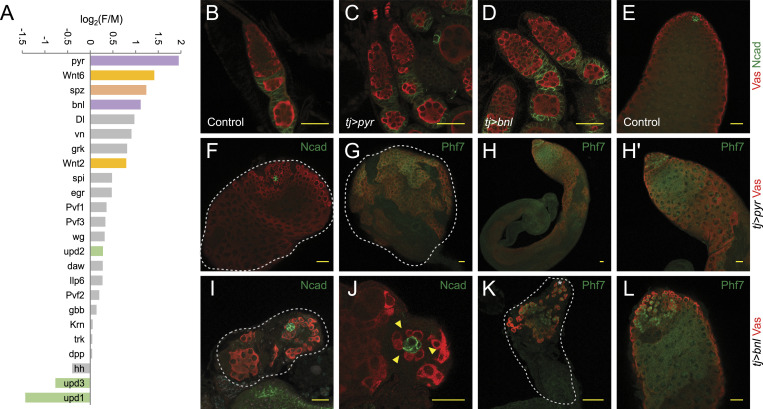
Sex-biased ligands in the embryonic somatic gonadal precursors (SGPs). **(A)** FSGP-to-MSGP expression ratios of signaling ligand–encoding genes plotted on a log scale; those whose read counts from these two clusters added together were less than 10 were excluded. Genes of ligands of the FGFR, Wnt, Toll, and JAK/STAT pathways are highlighted in purple, orange, rose, and green. **(B, C, D, E, F, G, H, I, J, K, L)** Gonadal phenotypes associated with FGF ligand overexpression in the somatic gonad revealed by immunofluorescence staining. **(B, C, D)** Ovaries of control (*tj-Gal4/+*, (B)), somatic *pyr* overexpression (*tj-Gal4/UAS-pyr*, (C)), and somatic *bnl* overexpression (*tj-Gal4/UAS-bnl*, (D)) genotypes stained with antibodies against Vas (red) and N-cadherin (green). **(E, F, G, H, I, J, K, L)** Testis images of control (*tj-Gal4/+*, (E)), somatic *pyr* overexpression (*tj-Gal4/UAS-pyr*, (F, G, H)), and somatic *bnl* overexpression (*tj-Gal4/UAS-bnl*, (I, J, K, L)) genotypes stained with antibodies against Vas (red) and N-cadherin (green) or Phf7 (green) as labeled on individual panels. **(H, H′)** is a magnified image of (H) around the testicular apex. **(F, G, I, K)** Areas encircled by white dotted lines indicate the entirety of abnormally small testes. All scale bars indicate 20 μm.

During embryogenesis, a key role that SGPs play in germline development is to provide the somatic sex information, which needs to correspond to the germline-intrinsic sex to nurture germline sexual development (Murray et al, 2010). The sex signal from the MSGP to the embryonic male germline is mediated by Unpaired (Upd) ligands to activate JAK/STAT signaling in the male germline (Wawersik et al, 2005), and when we examined the sex-biased expression of signaling pathway components in our scRNA-seq dataset for SGP populations, *upd1* exhibited the highest male bias, whereas *upd3* also showed male-enriched expression (Fig 6A).

The female somatic sex signal has not been identified, and our data suggested several promising candidates. Of the genes that encode signaling ligands, the one that exhibited the strongest female-biased expression in the somatic embryonic gonad was *pyramus* (*pyr*), which activates the FGFR pathway (Fig 6A). A second FGF ligand–encoding gene, *branchless* (*bnl*), also showed a clear female-biased expression (Fig 6A). Two other pathways that could be involved are Wnt and Toll signaling. The genes encoding *Wnt oncogene analog 2* (*Wnt2*) and *Wnt oncogene analog 6* (*Wnt6*) both ranked high on the female SGP-enriched list with *Wnt6* being second highest (Fig 5A). The third highest was *spatzle* (*spz*) whose encoded product is a ligand for the Toll pathway (Fig 5A). Interestingly, the FGFR and Wnt pathways have been shown to regulate ovarian development in later stages of the fruit fly life cycle (Irizarry & Stathopoulos, 2015; Wang & Page-McCaw, 2018), and it is quite possible that the same pathways also act sex-specifically at the early embryonic stages. These observations also suggested the possibility that ligands of multiple pathways collectively transmit the sex signal from the female soma to the germ cells.

To investigate whether the top female SGP-biased ligands were functionally significant in a sex-specific manner, we induced the ectopic expression of both FGF ligands of interest in the somatic gonad of both sexes. The overexpression of *pyr* and *bnl* in the female somatic gonad resulted in minimal-to-no defects (Fig 6B–D). We will note that in the case of *pyr* activation, ectopic expression of Vas occurred in the terminal filaments in ovaries, but there were no strong morphological defects otherwise in these gonads. Ovaries overexpressing *bnl* in the early soma were virtually identical as controls (Fig 6D versus Fig 6B). In comparison, the overexpression of either ligand in the early somatic gonadal compartment in the testes gave rise to strong morphological defects (Fig 6E–L). Phenotypes associated with *pyr* overexpression in the testicular soma were not fully penetrant, with many samples appearing to be undistinguishable from controls based on the morphology and marker expression (Phf7, Fig 6H and H′). However, a subset (11%) of the testes exhibited complete elimination of spermatids (Fig 6F and G); this phenotype disrupted the overall shape of the gonad, making it difficult to fully ascertain whether earlier stages of germline were normal apart from exhibiting the expected expression patterns for the germline stem cell and spermatogonial marker Phf7 (Fig 6G).

The overexpression of *bnl* in the male somatic gonad led to even stronger phenotypes in the testis though a fraction of these testes remained near-normal (Fig 6I–L). Of the testes that exhibited morphological defects, the majority had a loss-of-spermatogonia phenotype (55%, Fig 6I). Some of those further suffered from germline stem cell losses (18%, arrowheads, Fig 6J), though this defect could be secondary to the disruption of tissue homeostasis caused by spermatogonial loss. Yet, another phenotypic category observed was loss of spermatocyte (5%, Fig 6K), and this is reminiscent of the major phenotype observed with *pyr* overexpression. These analyses confirmed the sex-specific roles of the FGFR pathway in regulating germline development.

## Discussion

In this study, our major focus was to investigate how regulation at the chromatin level helps establish the germline program for zygotic activation and sex determination with the latter being initiated with the occurrence of the former (Li et al, 2021). Our scATAC-seq analysis indeed revealed aspects of chromatin structure unique to the germline. In addition, there are sex differences likely paving the way for germline sexual differentiation, one of which being a short transitory phase in the male germline right after zygotic activation. Chromatin accessibility in MGC1 appeared to be less structured, likely in part due to lower nucleosome occupancy, but within a few hours, the male germline transitioned out of these unusual chromatin states. Such transient and more “open” epigenetic status of MGC1 is reminiscent of epigenetic reprogramming in the early developing germline mammalian primordial germ cells known to undergo (Ramakrishna et al, 2021). Early *Drosophila* germline has not been shown to undergo similar processes to reset its epigenome, and what we report here could represent the *Drosophila* version of reprogramming its germline epigenome to facilitate future development of the unique germline program and to execute the correct sexual fate choice. There could be an equivalent process that occurs a few days later during the third-instar larval stage in the female germline as they initiate their development.

The exact molecular nature of the rapid epigenome reorganization requires future research to elucidate, but our analysis has implicated the pioneer factor Zld (Fig 3B), the key factor in the opening of chromatin accessibility in the early fruit fly embryonic soma. Zld activity in early embryogenesis is also necessary to secure proper germplasm deposition and early germline transcriptional quiescence, both of which are important for germline specification (Colonnetta et al, 2023). It is possible that Zld further acts during germline zygotic activation in a manner analogous to what happens during somatic zygotic activation. Interestingly, there was a subset in MGC1 that exhibited particularly high Zld binding site accessibility even though the average for MGC1 was not substantially higher than that for other clusters (Fig 3E). This could be pointing to a short time window during which Zld targets are being acted upon to allow male germline development. Furthermore, the emergence of M1BP and BEAF-32 as top candidates exhibiting differential germline activity brings forth the idea that they act as the antidote to Zld in keeping the genome quiescent, thereby explaining their elevated activity in the female germ cells after zygotic activation has occurred. Notably, Zld is known to work together with the GAGA factor (GAF) (Chetverina et al, 2021), yet another boundary element binding protein; the potential of Zld cooperating with other boundary element binding proteins such as M1BP or BEAF-32 to regulate germline development is clearly there.

Another important component of our data lies in the revelation of sex-biased factors in the embryonic gonad and germline. Germline sex determination requires two inputs, the intrinsic sex chromosome content from the germline and an extrinsic signal from the surrounding SGPs, and these two need to match for germ cells to reach a successful sex choice (Murray et al, 2010). The identity of the male SGP signal to male germline occurs through the JAK/STAT pathway, but neither the female SGP signal nor the mechanisms of how germ cells read their own sex have been elucidated. Our comprehensive coverage of the embryonic germline has been able to provide strong candidates for the latter two mechanisms. For the female SGP-to-germline sex signal, FGF ligands Pyr and Bnl were at the top of the candidate list, though genes encoding two Wnt ligands also showed strong female-enriched expression (Fig 6A). This is in contrast to the genes encoding Upd ligands being virtually the only ones showing male-enriched expression. Indeed, our overexpression experiments with *pyr* and *bnl* resulted in sex-specific phenotypes, but the phenotypes were not as severe as one would expect if these ligands acted as the dominant somatic sex signals (Fig 6B–L). One possible scenario for the female somatic sex signal is that it is consisted of both FGFR and Wnt signaling, potentially in a redundant manner. This could also help explain why the female signal has been more difficult to identify. A similar combinatorial mechanism could also be in place for how germ cells read out their sex chromosome composition intrinsically, and the genes involved could be those that exhibit the earliest signs of sex-biased expression. Our 0- to 8-h scRNA-seq data found them to be a set of female-biased ones on the X chromosome with the majority encoding TFs or proteins predicted to have DNA binding abilities (Li et al, 2021). It is quite conceivable that more than one of these factors functions cooperatively as the germline-autonomous sex-determining switch.

## Materials and Methods

### Fly strains

For scRNA-seq and scATAC-seq experiments, *vas-GFP (II)*; *vas-GFP (III)* flies were used (Shigenobu et al, 2006). For sexed embryonic staining, *P{Sxl-Pe-EGFP.G}G78b* (*Sxl-GFP*) embryos were used for the stage 9–10 time points, whereas to obtain stage 17 samples, *w*^*111*8^ virgins were crossed with *FM7c,P{Dfd-GMR-nvYFP}1* males. For RNAi knockdown experiments, *nos-Gal4* flies (Van Doren et al, 1998) were crossed with *P{TRiP.HMJ22253}attP40/CyO* for *CG18605*; *P{UAS-GFP.nls}14* (*UAS-GFP*) was used as the control. For overexpression experiments, *traffic jam (tj)-Gal4* (Li et al, 2003) flies were crossed with *P{UAS-pyr.K}/TM3, Sb*^*1*^*, Ser*^*1*^, and *P{UAS-bnl.S}A12/CyO* flies to drive the SGP expression of *pyr* and *bnl*, respectively. Strains were obtained from the Bloomington Stock Center unless otherwise noted.

### Sample preparation and multiome data acquisition

Embryos for scRNA-seq were collected in two time windows, 8–12 and 12–20 h. All embryos of the designated ages were dechorionated, Dounce-homogenized gently with 7–15 strokes, filtered through 40-μm mesh, pelleted at 850*g* for 2 min, and FACS-sorted to obtain germ cells (8–12 h) or gonads (12–20 h). For gonads, single germ cells and SGPs were obtained by dissociation for 15 min in 0.25% trypsin and 1 mg/ml collagenase. Cell numbers for the two samples were counted under light microscopy, pooled, and sent for scRNA-seq.

Nucleus preparations followed the protocol for single germ cell and gonad isolation with the addition of cell lysis steps at the end in lysis buffer (10 mM Tris, pH 7.4, 10 mM NaCl, 3 mM MgCl_2_, 1% BSA, 0.01% Tween-20, 1 mM DTT, 0.01% NP-40, 0.001% digitonin, and 1 U/μl Protector RNase inhibitor from Sigma-Aldrich) at 4°C. Sample collection was broken into three time windows, 0–4, 4–12, and 12–20 h, as their optimal lysis times differed at 5, 4.5, and 5.5 min, respectively. Nuclei were then washed and resuspended in wash buffer (10 mM Tris, pH 7.4, 10 mM NaCl, 3 mM MgCl_2_, 1% BSA, 0.1% Tween-20, 1 mM DTT, and 1 U/μl Protector RNase inhibitor from Sigma-Aldrich) before counting under a light microscope and pooling of samples and submission for multiome analysis that included scATAC-seq and snRNA-seq.

Both scRNA and multiome sequencing were performed commercially (BioTools), which included sample quality control, library construction, and high-throughput sequencing on the 10x Genomics platform following the manufacturer’s protocol and using standard parameters.

### Data analysis

Our previous scRNA-seq data from 0- to 8-h embryonic germline were combined with those from the 8- to 20-h dataset for comprehensive analysis of germline gene expression with a total of 16,371 cells being analyzed. Sequencing reads from the combined datasets were aligned to the *D. melanogaster* reference genome (BDGP6.28.102) and aggregated using CellRanger v6.1.2. Clustering was performed with Monocle 3 (Cao et al, 2019) using PCA and UMAP for preprocessing and dimension reduction, respectively, and with a dimensionality reduction value of 150. Differential gene expression analysis between different clusters was performed with the DESeq2 package (Love et al, 2014), and the output files excluded genes whose total counts between the two clusters being compared were less than 10. For calculating expression ratios for motif discovery, a cutoff of 1 was set for snRNA-seq expression values for germline clusters, whereas 0.01 was added to all soma GRO-seq values as calculated previously (Li et al, 2021) to avoid dividing by zero values. For examining dosage compensation at the gene expression level, the set of 345 zygotically activated genes (Table S4) was selected based on their average expression data in MGC2 and FGC2 compared with GC using DESeq2 and applying the cutoffs of fold differences >2 and *P* < 10^−10^. Discovery of common motifs enriched in marker genes of germline clusters was performed with MEME (Bailey et al, 2009) by taking the 500-bp regions upstream of TSSs of the 150 genes exhibiting the strongest expression enrichment in the MGC1, MGC2, FGC1, and FGC2 clusters of the snRNA-seq dataset compared with values of the same genes from the GRO-seq data for embryonic soma.

The processing of our scATAC-seq data began with the alignment of reads to the *D. melanogaster* reference genome (BDGP6.28.102) and assignment of reads into different cells based on the barcode sequences using CellRanger ARC v1.0.0. To eliminate low-quality cells, a series of filtering rules were applied. First, empty droplets were removed using the DropletUtils package (Lun et al, 2019). Second, cells with a nuclear RNA count lower than 1,000 were discarded. Third, double or triple droplets were identified and removed using the scDblFinder package (Germain et al, 2021). Last, low-quality cells that matched one of the following criteria were filtered out by Signac (Stuart et al, 2021): ATAC counts lower than 100 or higher than 100,000, TSS enrichment score higher than 1, nucleosome signal lower than 2, or a percentage of mitochondrial genes lower than 40. This resulted in a total of 3,022 nuclei that passed quality control and were further analyzed using the following features in the Signac package. Counts of snRNA data were normalized using the SCTransform method with the number of variable features and dimension reduction value set to 5,000 and 150, respectively. ATAC counts were calculated using the embedded MACS2 function and subjected to normalization and dimensional reduction with the value set at 150 using the LSI method. The snRNA-seq and scATAC-seq datasets were then integrated to construct a WNN graph, which enabled clustering and the designation of cell types based on expression patterns of cell-type marker genes. To assess accessibility of binding motifs of TFs, scATAC-seq read data were analyzed using the chromVAR function for all TFs in the JASPAR database (version: 2020). Overall differences in TF site accessibility between clusters were subsequently analyzed by taking average accessibility of all binding sites for PCA using the PCAtools package. To describe the accessibility pattern over the lengths of genes, ATAC-seq reads located within 250 bp upstream and downstream of either the 3′ end or TSSs of genes were counted, and normalized to sizes of clusters before being divided with each other to obtain end-to-start accessibility (ESA) scoresfor individual clusters. Sex-biased expression in the adult germline was calculated using expression values in adult ovaries and testes from the Fly Cell Atlas database (Li et al, 2022).

### Immunofluorescence

Staining of embryos followed previous protocols with the modification of detergent added (Yang et al, 2012); for stage 17 stains, 0.3% Triton X was used, whereas for stage 9–10 stains, 0.1% Tween-20 was used. Larval and adult gonad stains also followed previously published procedures (Yang, 2021). Antibodies used included rabbit α-histone H3 (1:100, PA5-16183; Invitrogen), rat α-N-cadherin (1:20, DN-Ex#8; DHSB), rabbit α-Phf7 (1:2,500) (Yang et al, 2017), and Alexa Fluor–conjugated secondary antibodies (1:500; Jackson ImmunoResearch). Antibodies against Vas were generated by expressing amino acids 1–260 of the *D. melanogaster* Vas protein with a His-tag in bacteria using the pET-28 vector followed by immunization of the purified protein in rabbit and guinea pig hosts. The resulting rabbit α-Vas and guinea pig α-Vas antibodies were both used at 1:1,000.

To quantitate histone H3 signals, average histone H3 intensities within each nucleus were calculated and then divided by the average DAPI intensity of the same area. To further correct for staining efficiency differences between embryos, the aforementioned normalized H3 signals in germ cells were divided by average H3 signals of all SGPs within the same embryonic gonad. Larval germline stem cells were determined as those adjacent to terminal filaments in ovaries and hubs in testes, both of whose structures were identified by N-cadherin staining. Images were taken on a confocal microscope (LSM780; Zeiss).

## Supplementary Material

Reviewer comments

## Data Availability

All raw sequencing and processed count data generated for this study are available at the NCBI Gene Expression Omnibus under the accession number GSE240043.
