# Supplementary material for: Transient chromatin decompaction at the start of D. melanogaster male embryonic germline development
Source: Life Sci Alliance. 2024 Jul 11;7(10):e202302401. doi: 10.26508/lsa.202302401 (PMC11239976; doi:10.26508/lsa.202302401)
Supplement: Supplementary file 2 [file LSA-2023-02401_TableS2.docx]

**Table S2.** Common motifs discovered in genes enriched at the transcriptional level in the indicated germ cell populations post zygotic activation.

| Clusters^a,b^ | | | | TF Candidate |
| --- | --- | --- | --- | --- |
| FGC1^c^ | FGC2^c^ | MGC1^c^ | MGC2^c^ |  |
| 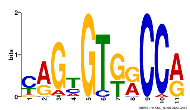  1.3x10^-50^ | 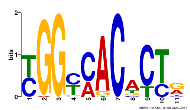1.6x10^-45^ | 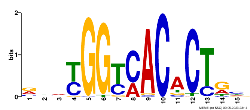1.5x10^-61^ | ^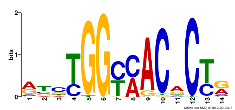^4.6x10^-40^ | M1BP |
| 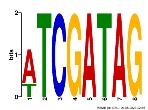  6.4x10^-10^ | 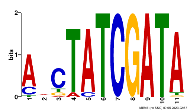4.1x10^-6^ | 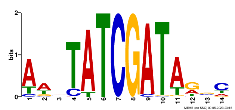1.6x10^-15^ | 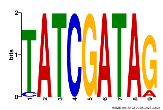  7.3x10^-3^ | BEAF-32/  Dref/  Pnr |
| 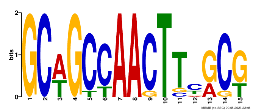  1.4x10^-3^ |  |  |  | CG4854 |
|  |  | 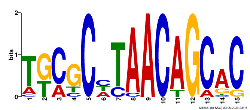4.5x10^-7^ | ^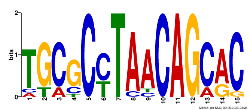^9.6x10^-4^ | Caup |
|  | 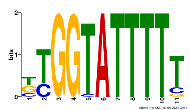1.2x10^-2^ | 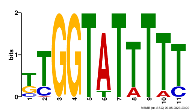  2.0x10^-5^ |  | CG11352 |
|  |  | 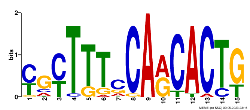5.3x10^-3^ |  | CG7928/  CG10267 |

^a^The clusters here refer to those from the snRNA-seq dataset.

^b^Top 150 most enriched genes for each cluster compared to the gene body signal from the GRO-seq reference dataset were used.

^c^The E-values are indicated beneath each motif; a cutoff of <5x10^-2^ was used.
